# Feasibility of regional center telehealth visits utilizing a rural research network in people with Parkinson’s disease

**DOI:** 10.1017/cts.2024.498

**Published:** 2024-03-25

**Authors:** Tuhin Virmani, Lakshmi Pillai, Veronica Smith, Aliyah Glover, Derek Abrams, Phillip Farmer, Shorabuddin Syed, Horace J. Spencer, Aaron Kemp, Kendall Barron, Tammaria Murray, Brenda Morris, Bendi Bowers, Angela Ward, Terri Imus, Linda J. Larson-Prior, Mitesh Lotia, Fred Prior

**Affiliations:** 1 Department of Neurology, University of Arkansas for Medical Sciences, Little Rock, AR, USA; 2 Department of Biomedical Informatics, University of Arkansas for Medical Sciences, Little Rock, AR, USA; 3 Translational Research Institute, University of Arkansas for Medical Sciences, Little Rock, AR, USA; 4 Rural Research Network, University of Arkansas for Medical Sciences, Little Rock, AR, USA; 5 Regional Programs, University of Arkansas for Medical Sciences, Little Rock, AR, USA; 6 Department of Biostatistics, University of Arkansas for Medical Sciences, Little Rock, AR, USA; 7 Institute for Digital Health and Innovation, University of Arkansas for Medical Sciences, Little Rock, AR, USA

**Keywords:** Telemedicine, health equity, rural health, ambulatory monitoring, Parkinson’s disease, medically underserved area

## Abstract

**Background:**

Impaired motor and cognitive function can make travel cumbersome for People with Parkinson’s disease (PwPD). Over 50% of PwPD cared for at the University of Arkansas for Medical Sciences (UAMS) Movement Disorders Clinic reside over 30 miles from Little Rock. Improving access to clinical care for PwPD is needed.

**Objective:**

To explore the feasibility of remote clinic-to-clinic telehealth research visits for evaluation of multi-modal function in PwPD.

**Methods:**

PwPD residing within 30 miles of a UAMS Regional health center were enrolled and clinic-to-clinic telehealth visits were performed. Motor and non-motor disease assessments were administered and quantified. Results were compared to participants who performed at-home telehealth visits using the same protocols during the height of the COVID pandemic.

**Results:**

Compared to the at-home telehealth visit group (*n* = 50), the participants from regional centers (*n* = 13) had similar age and disease duration, but greater disease severity with higher total Unified Parkinson’s disease rating scale scores (*Z* = −2.218, *p* = 0.027) and lower Montreal Cognitive Assessment scores (*Z* = −3.350, *p* < 0.001). Regional center participants had lower incomes (Pearson’s chi = 21.3, *p* < 0.001), higher costs to attend visits (Pearson’s chi = 16.1, *p* = 0.003), and lived in more socioeconomically disadvantaged neighborhoods (*Z* = −3.120, *p* = 0.002). Prior research participation was lower in the regional center group (Pearson’s chi = 4.5, *p* = 0.034) but both groups indicated interest in future research participation.

**Conclusions:**

Regional center research visits in PwPD in medically underserved areas are feasible and could help improve access to care and research participation in these traditionally underrepresented populations.

## Introduction

Clinical care from a neurologist has been shown to improve outcomes in People with Parkinson’s disease (PwPD)[1]; however, access to specialty care remains a significant issue. Motor, cognitive, and visuospatial impairment in PwPD can lead to limitations in driving [2-4], especially for longer distances, or in less familiar areas. We previously showed that at-home telehealth visits can make PwPD feel more self-reliant in their care despite the increased technological knowledge needed to complete such visits [5]. Travel distance can also often deter research participation, and we previously showed that PwPDs given the opportunity for telehealth-based research were more likely to participate in future research studies[5].

**Figure 1.**
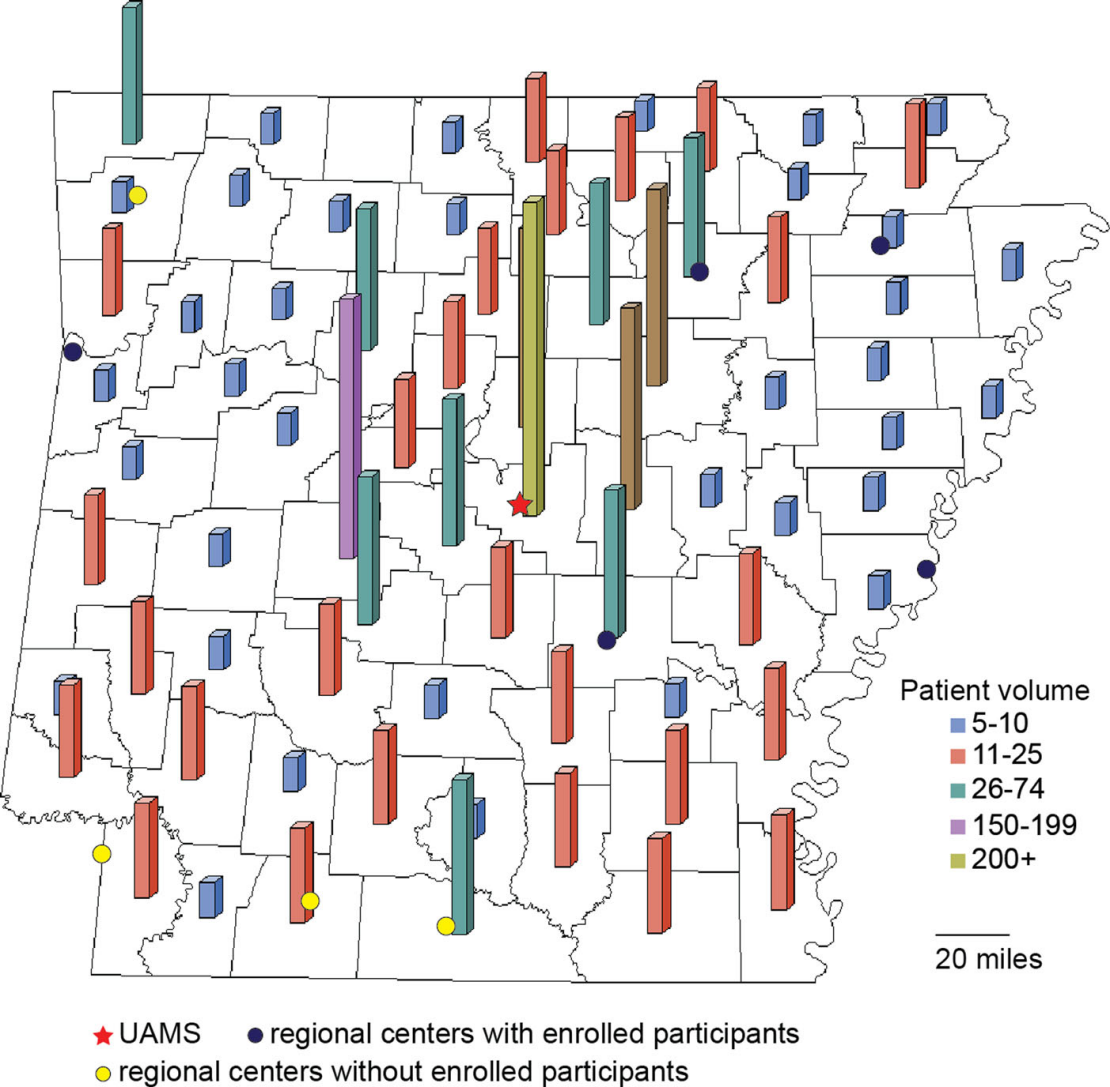
Distribution of people with Parkinson’s disease living in medically underserved areas in Arkansas who obtain their clinical care at the University of Arkansas for Medical Sciences (UAMS) movement disorders clinic. The star indicates the location of UAMS’ main campus in Little Rock while the circles depict the locations of the UAMS regional centers (rural research network) around the state. Despite the higher socioeconomic status of central and NW Arkansas, a significant portion of the medically underserved population cared for at the UAMS MDC resides in central Arkansas. However, PwPD residing in medically underserved areas cared for at UAMS are scattered around the state and located in areas with clusters of underserved people around them.

In a rural state such as Arkansas, access to care can be especially difficult for PwPD. In Arkansas, 4 of the 5 movement disorders fellowship-trained neurologists practice at a single institution, the University of Arkansas for Medical Sciences (UAMS). Approximately 50% of the PwPD obtaining clinical care at the UAMS Movement Disorders Clinic (MDC) reside in designated medically underserved areas (MUAs) and are scattered around the state (Fig. 1). Over 70 and 40% of the UAMS MDC patients travel over 30 and 60 miles respectively to obtain clinical care in-person.

Objective, secure, and reliable methods of tracking disease progression closer to home via telemedicine in people with limited access or comfort with technology could improve access to care and mitigate some of the costs of care [6,7], even though they may not completely replace in-person care [8].

The COVID-19 pandemic led to a widely increased utilization of telehealth for clinical evaluations in movement disorders [9]. While most patients and physicians remain satisfied with this mode of care delivery, several concerns have also been raised regarding continued widespread adoption of digital technology for clinical care [5,8-10]. Of these, limited cellular or high-speed internet connectivity and low socioeconomic status are often cited as factors leading to decreased access to care by adoption of telehealth [11,12], thereby widening the so-called digital divide. Based on data from 2018 approximately 38% of older adults were estimated to be unready for video visits, predominantly related to inexperience with technology [13]. This would be applicable to the population of PwPD and attempts to use new technology could lead to more frustration instead of improved patient outcomes. Additionally, approximately 41.4% of Medicare beneficiaries lacked access to a desktop or laptop computer with high-speed internet connectivity and a similar percentage (40.9%) also lacked access to a smartphone with a wireless data plan [14]. In Arkansas, while significant inroads are being made, a large percentage of the state’s population does not have access to broadband internet access [15].

There are also limitations related to the nature of a video visit, and the inability to “lay hands” on a patient during a clinical exam. While the majority of the Unified Parkinson’s disease rating scale can be performed even by reviewing videos of research participants, two core features of rigidity and postural instability require examiners to be present with the patient. However, a recent study found that the same number of DAT scans were ordered during telehealth visits of new patients compared to in-person visits, suggesting that the lack of rigidity and postural instability measures may not have impacted parkinsonism diagnosis significantly [16]. We also previously showed that at-home assessments in Phase 1 of the current study, performed during the peak of the COVID-19 pandemic, not only had high participant satisfaction, ability to recruit participants from medically underserved areas, and high interest in future participation in telehealth research, but that in those with previous research visits, the results of motor and non-motor assessments were comparable [5].

To overcome the limitations of those who are unable to obtain telehealth care at home, a hub-and-spoke model of care could be utilized. People could be seen at a local regional clinic closer to their homes, using telehealth resources unavailable to them at home, thereby decreasing travel burden. Such models have been successfully employed in other neurologic diseases, with acute stroke care being a great example of the improved patient-centered outcomes. Our goal in this study was therefore to determine the feasibility of performing telehealth-based clinic-to-clinic video visits in PwPD at regional centers closer to their residence. To achieve these goals, we utilized the UAMS rural research network [17], which leverages 10 UAMS family medicine clinics around the state of Arkansas, to perform clinic-to-clinic telehealth visits in PwPD. Our hypothesis was that PwPD volunteering to participate in regional clinic telehealth-based clinical/research visits would have greater disease burden and lower socioeconomic status than those who previously participated in at-home telehealth clinical/research visits.

## Materials and methods

### Standard protocol approvals, registrations, and patient consents

Participants who had been previously seen for a diagnosis of Parkinson’s disease at the Movement Disorders Clinic (MDC) at the University of Arkansas for Medical Sciences (UAMS) and resided within 30 miles of one of the UAMS regional center clinics were recruited for this study. The regional center clinics were in Batesville, Fort Smith, Pine Bluff, Jonesboro, and Helena, Arkansas. Approval for the study was obtained from the UAMS institutional review board (IRB#261021).

Potential participants who were prescreened for meeting the above criteria were approached, the study was explained in detail, and consent forms were provided for review. Potential participants were then contacted again and if agreeable to participate, scheduled for a visit at the regional center near them at their convenience. Study visits were performed between October 2021 and June 2022. All participants were evaluated at a UAMS regional center clinic using clinic-to-clinic telemedicine using a CISCO weblink for secure connectivity. A telehealth cart including a Dell 3090 minicomputer and the CISCO room kit mini (microphone, camera, and speakers) was provided to each regional center clinic by the UAMS Institute for Digital Health and Innovation.

Regional center nurses were trained in the performance of orthostatic vitals. The UAMS Rural Research Network research coordinators provided onsite assistance to participants in the consent process and in use of technology to complete assessments as needed. The research coordinators were guided through the assessments by trained research personnel (LP and AG) with previous experience in administering the assessments. The regional center nurses and Rural research network coordinators were trained on the use of the telehealth equipment by UAMS Information Technology (IT) personnel. Each regional center site was provided an opportunity to ask questions to the principal investigator and UAMS site team prior to the visit and IT personnel were available to help solve technical issues during the visit.

### Comparison group

This study was initially designed to enroll PwPD either at home or at a regional center during the same period. However, due to the COVID-19 pandemic, the first set of 50 participants were all evaluated at home as the regional centers were closed. The results of this initial group or Phase 1 of the study were previously published [5]. As initially intended, the at-home participants from Phase 1 are used as a comparison group for the participants from the regional centers (now study Phase 2) reported in this manuscript.

### Study assessments

Instruments for remote administration of study assessments were created in the Research Electronic Data Capture database (REDCap). The methods for deployment of these assessments were previously reported in detail [5]. Briefly, standard of care assessments included a clinical history of participants’ Parkinson’s disease, medication and allergy profile, orthostatic vitals, administration of a previously validated modified version of the Unified Parkinson’s Disease Rating Scale (UPDRS) [18] that excludes the motor assessments of tone (UPDRS item 22) and balance (UPDRS item 30) and a remotely administered Montreal Cognitive Assessment (MoCA) [19]. For the MoCA, the visuospatial tasks were displayed on the participants' televideo screen via screen share, and results were again obtained immediately via the video feed and scanned copies mailed back to us.

Research assessments performed included the new freezing of gait questionnaire (N-FOGQ) [20], handwriting samples on a preprinted sheet with instructions, gait using the Timed Up and Go test (TUG), voice samples using a secure voicemail, the Parkinson’s disease quality of life scale-39 (PDQ-39) [21], the Epworth sleepiness scale (ESS) [22], and the REM sleep behavior disorder questionnaire (RBD-Q) [23]. Participants were also asked to complete a survey gauging their perception of audio-video quality and visit satisfaction. It was optional for them to provide their annual income range and estimated costs to attend in-person visits. The research team also assessed audio-video quality, perceived issues, and relative time to perform assessments over telemedicine compared to in-person.

### Socioeconomic status measures

Residence addresses of participants were used to obtain their Area Deprivation Index (ADI) status using the online tool provided by the University of Wisconsin website [24,25]. The ADI uses factors such as income, education, employment, and housing quality to help rank neighborhoods by socioeconomic disadvantage. Both national percentiles and Arkansas state-based deciles were used to compare participants in this study.

### Statistical analysis

Statistical analysis was performed using SPSS version 25 (IBM). Normality was tested using the Shapiro-Wilk test for each assessment. Due to the number of non-normal distributions, the Mann-Whitney *U*-test (MW) was used to compare groups for continuous variables while the Pearson’s chi-square test was used for nominal variables.

### Data sharing

All study data from the current collection (Phase 2) and prior Phase 1 collection were combined into a single collection using the Arkansas Research Image Enterprise System (ARIES) [26,27]. ARIES supports integration of multimedia data, including sound files, and extracts from both the REDCap database and the UAMS Arkansas Research Clinical Data Repository (AR-CDR) [28,29]. All ARIES data are de-identified using an integrated utility [29]. Study data will be made available upon publication of the study.

## Results

Thirteen PwPD were enrolled for visits from 5 of the UAMS regional centers in this phase of the study located west, northeast, south, and east of Little Rock (Fig. 1, star and black circles). The results of the regional center participants were compared with 50 PwPD previously enrolled in at-home visits in phase 1 of the study during the COVID-19 pandemic. Both groups had similar ages at enrollment and sex distribution (Table 1). Participants had similar disease duration in both groups (Table 1; 10.0 ± 5.5 vs 9.2 ± 5.7 years; regional center vs. at-home; Mann-Whitney *U* (MW) *Z* = −0.5, *p* = 0.599), but participants performing visits at the regional centers had greater disease severity with higher Hoehn and Yahr staging scores (MW *Z* = −2.2, *p* = 0.026), higher total Unified Parkinson’s Disease rating scale scores (MW *Z* = -2.2, *p* = 0.027) and lower Montreal Cognitive Assessment scores (MW *Z* = −3.4, *p* < 0.001) than at-home participants (Table 1). The regional center participants also endorsed a worse quality of life than at-home participants (MW *Z* = −2.149, *p* = 0.032). On objectively quantified measures, regional center participants had a slower TUG performance time (MW *Z* = −3.270, *p* = 0.001) and smaller spirals in both the dominant (MW *Z* = −2.208, 0.027) and non-dominant hands (MW *Z* = −2.157, 0.031) (Table 1). Voice samples were trimmed for silence before and after the Ah sound. Samples that were less than 1.5 s after trimming were excluded from the analysis. The remaining trimmed samples (9 regional centers, 40 at-home) did not show significant group differences in any of the primary measures of voice (Supplementary Table 1).

**Table 1.** Participant demographics and results of clinical and research assessments

	Regional center participants (*n* = 13)	At-home participants (*n* = 50)
Sex (Female/male)	8/5	30/20
Education (years)	12.9 ± 1.9*	16.3 ± 2.4
Race (Caucasian %)	12 (92%)	50 (100%)
Reside in MUA	10 (77%)^#^	20 (40%)
Age at enrollment (years)	70.4 ± 6.9	65.8 ± 9.2
Disease duration (years)	10.0 ± 5.5	9.2 ± 5.7
Distance from UAMS (miles)	115 ± 41*	60 ± 63
Distance to Regional center (miles)	25 ± 27	
Travel distance saved (miles)	90 ± 42	
No prior research participation	77%^#^	44%
Motor features:		
Hoehn & Yahr stage	2.3 ± 0.4	2.0 ± 0.5
Modified motor UPDRS	16.7 ± 8.5	12.5 ± 6.4
Modified total UPDRS	32.9 ± 13.8*	24.0 ± 10.7
Freezing of gait (FOG)	10 (77%)^#^	17 (34%)
Non-motor features:		
MoCA score	21.7 ± 4.9*	26.1 ± 2.9
PDQ-39 score	49.0 ± 35.0*	27.5 ± 21.3
RBD-Q score	5.9 ± 3.2	5.0 ± 3.1
Epworth Sleepiness Scale score	8.8 ± 4.8	7.7 ± 4.9
Medications:		
Daily levodopa dose (mg)	596 ± 350	662 ± 328
On agonist/MOA-I	40%/40%	28%/40%
Objective measures:		
10 ft TUG Mean Time (s)	20.6 ± 17.0*	11.9 ± 3.1
Trial to trial variability (CV)	12.6 ± 9.6	7.0 ± 6.7
Spiral area - more affected hand (cm^2^)	60.7 ± 29.9*	83.8 ± 39.0
Spiral area - less affected hand (cm^2^)	65.0 ± 39.3*	90.3 ± 46.1

Values reported as mean ± stdev. *p*<0.05 by *Mann-Whitney *U*-Test or ^#^Chi-square test. MoCA = Montreal Cognitive Assessment; MOA-I = mono-oxidase inhibitor; MUA = medically underserved areas; PDQ = Parkinson’s disease quality of life scale; RBD-Q = REM sleep behavior disorder questionnaire; TUG = Timed-up-and-go test; UAMS = University of Arkansas for Medical Sciences; UPDRS = Unified Parkinson’s disease Rating Scale.

### Participant satisfaction survey

Table 2 shows results of a post-visit survey completed by participants. Overall participant satisfaction with the regional center visits was high (85% vs 92%; regional center vs at-home respectively), and ability to participate in research was a positive feature of the visits (69% vs 82%; regional center vs at-home). Only 1 participant in the regional center group preferred in-person visits compared to 14 (28%) of the at-home participants. Importantly, participants in both groups were more likely to participate in telemedicine research in the future after their experience (85% vs 62%; regional center vs at-home).

**Table 2.** Participant satisfaction survey results

	Regional center participants (*n* = 13)	At-home participants (*n* = 50)
Scheduling appointment was easy: Strongly agree	77%	86%
Somewhat agree	23%	14%
Neither agree nor disagree	0%	0%
Somewhat disagree	0%	0%
Strongly disagree	0%	0%
I was happy with my telehealth visit:	(n = 13)	(n = 49)
Strongly agree	85%	92%
Somewhat agree	15%	6%
Neither agree nor disagree	0%	0%
Somewhat disagree	0%	2%
Strongly disagree	0%	0%
What did you like about the telehealth visit:		
No travel arrangements	85%	70%
Ability to be in comfort of your home	23%	84%
Ability to participate in research	69%	82%
What did you dislike about the telehealth visit:		
Poor video connection	0%	6%
Unable to hear provider	15%	22%
Poor internet connection	15%	6%
Prefer in-person visit	8%	28%
More likely to participate in telehealth research in the future		
Strongly Agree	39%	29%
Somewhat Agree	46%	33%
Neutral	15%	33%
Somewhat Disagree	0%	2%
Strongly Disagree	0%	4%
Whom do you rely on for in-person visits? (check all that apply)		
Self	39%	64%
Spouse	54%	52%
Children	46%^#^	6%
Spouse	15%	2%
Whom did you rely on for telehealth visit? (check all that apply)		
Self	46%^#^	80%
Spouse	54%	34%
Children	31%^#^	2%
others	15%	6%
Overall visit rating: extremely bad	0%	0%
bad	0%	0%
neutral	0%	0%
good	8%	27%
excellent	92%	73%
Annual Income:	(n = 10)	(n = 43)
<$25,000	50%*	5%
$25–50,000	40%	16%
$50–75,000	10%	16%
$75–100,000	0%	14%
>$100,000	0%	49%
Costs to attend in-person visit:	(n = 10)	(n = 43)
<$35	0%*	56%
$36–75	60%	19%
$76–150	40%	12%
$151–300	0%	9%
>$300	0%	5%

*p* < 0.05 by *Mann-Whitney *U*-Test or ^#^Chi-square test.

### Socioeconomic status of participants

Participants from the regional centers had lower education levels (MW *Z* = −3.9, *p* < 0.001) than at-home participants (Table 2). They also had lower income distribution (MW *Z* = −4.155, *p* < 0.001) but higher costs to attend in-person clinic visits (MW *Z* = −2.201, *p* = 0.028) (Table 2). A higher percentage of participants from regional centers resided in designated medically underserved areas (MUAs) (Table 1) (Pearson’s chi-square = 5.6, *p* = 0.019).

**Figure 2.**
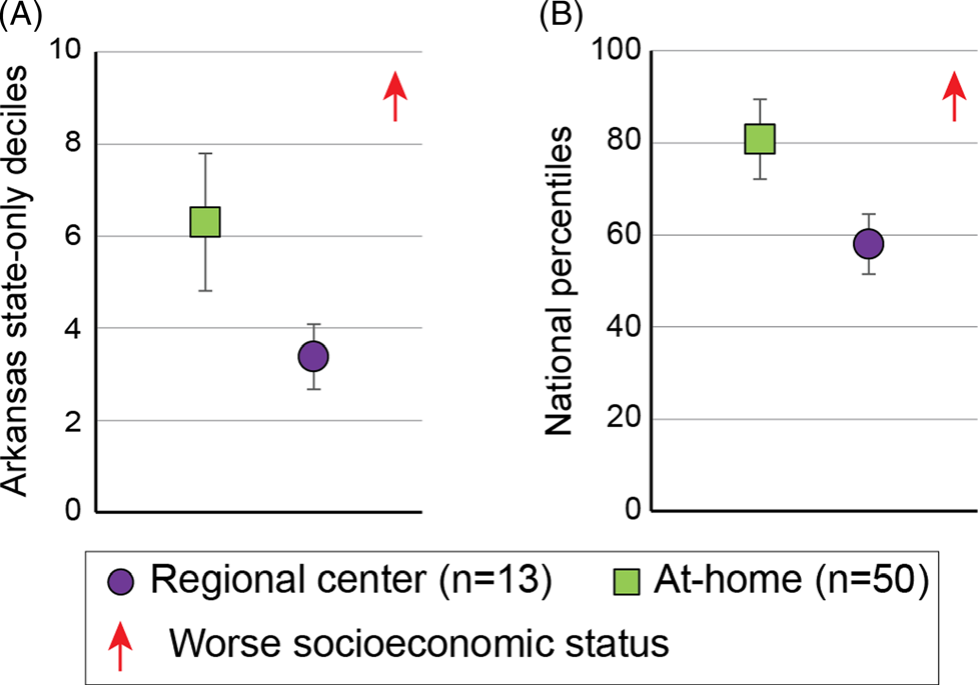
Area deprivation index of participants. Distribution of Area Deprivation Index (ADI) scores of study participants using (A) Arkansas state-only deciles and (B) national percentiles for regional center (green square) and -at-home (purple circle) participants. Results are plotted as means with 95% confidence intervals.

Regional center participants were on average at the 5.5 ± 2.3 decile for AR state and 76.6 ± 13.3 percentile nationally on the ADI index indicating residence in a more socioeconomically disadvantaged neighborhood than 75% of the US population and 55% of Arkansas’ population. The regional center participants also had higher Arkansas state-only (MW *Z* = −3.120, *p* = 0.002) and national (MW *Z* = −3.254, *p* = 0.001) ADI indices (Fig. 2), than the at-home group.

**Figure 3.**
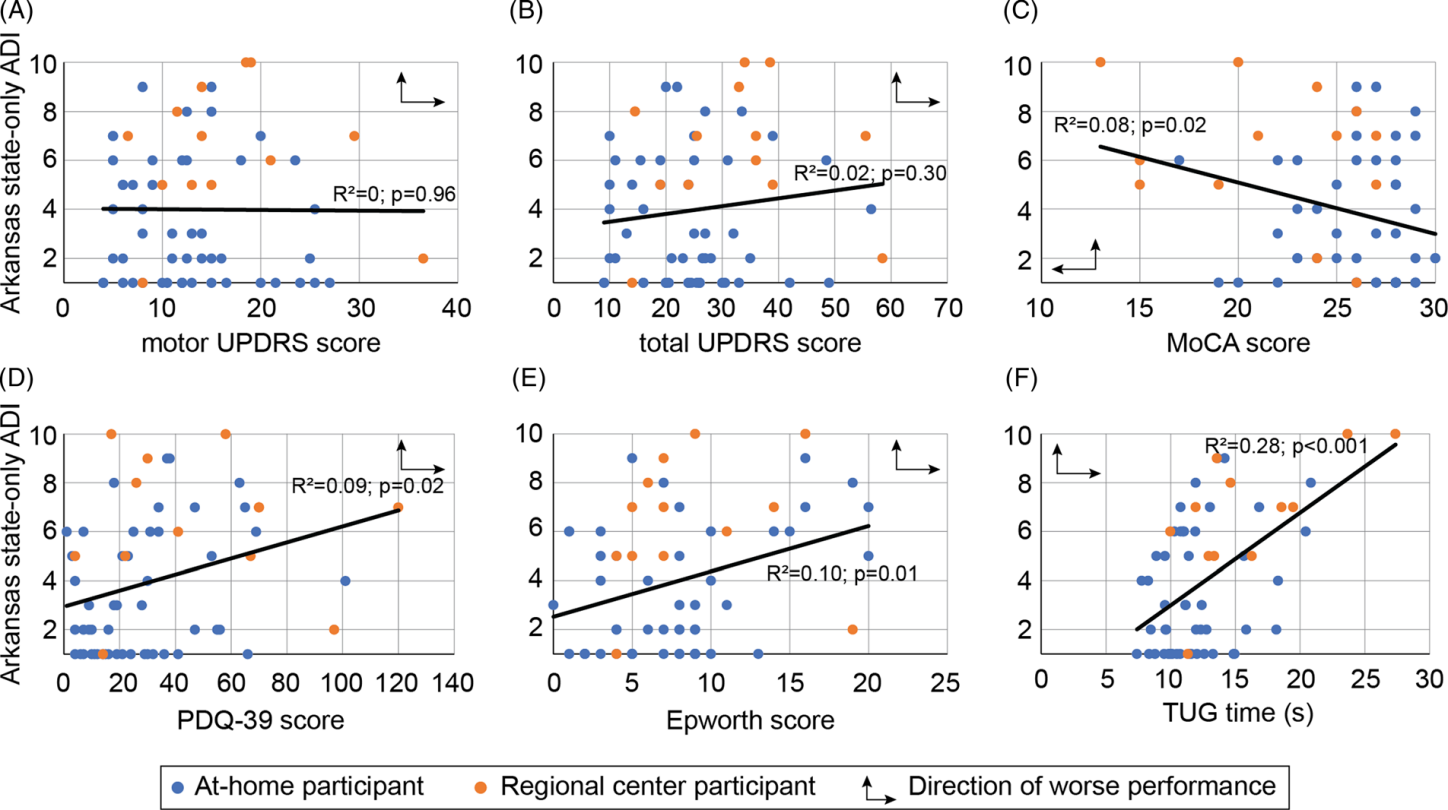
Area deprivation index compared to disease measures. Scatter plots of Arkansas state-only Area Deprivation Index (ADI) scores compared to participant (A) motor and (B) total Unified Parkinson’s Disease Rating Scale (UPDRS) scores, (C) Montreal Cognitive Assessment scores, (D) quality of life scores, (E) Epworth Sleepiness Scale scores, and (F) time to complete the Timed Up and Go (TUG) task. Blue circles denote at-home participants while orange circles denote regional center participants. Linear regression lines are plotted for the entire population. Arrow direction indicates worse performance on the assessment.

The relationship between socioeconomic status and disease severity for the different assessments performed is plotted in Fig. 3. The strongest association was between ADI score and time on the TUG task (Fig. 3F) but there was also a weak association with MoCA scores (Fig. 3C), PDQ-39 scores (Fig. 3D), and Epworth scores (Fig. 3E). Of note, there was no association between ADI score and motor and total UPDRS scores (Fig. 3A, B).

### Travel burden

Participants at the regional centers lived further away from UAMS (Table 1; 115 ± 41 vs. 60 ± 63 miles; regional center vs at-home; MW Z −3.3, *p* = 0.001) (Table 1) than at-home participants and were more reliant on their children for in-person visits to the UAMS MDC (Table 2; 46% vs 6 %; regional center vs at-home; Pearson’s chi-square *p* < 0.001)). Participating in clinical visits at local regional centers saved these participants on average 90 miles of travel distance one-way compared to driving into the UAMS MDC (Table 1).

### Comparison of visit quality

We also utilized a post-visit survey completed by our research group after each visit, to determine the audio-video quality, difficulties with performing assessments, and extra time needed for completion of assessments (Table 3). There were no significant differences in overall time required to setup or administer the standard of care assessments, or ability to perform specific assessments. Audio-video quality was rated slower in the at-home group. Participants had more difficulty performing the survey-based assessments (PDQ-39, RBD-Q, ESS, post-visit survey) in the regional center group. Overall, the regional center participants took over 30 minutes longer than the at-home participants to complete the visits (Table 3; MW *Z* = −4.630, *p* < 0.001).

**Table 3.** Research staff survey

	Regional center participants (n = 13)	At-home participants (n = 50)
Total visit time (hours)	2.1 ± 0.3*	1.4 ± 0.4
Extra time required to setup clinic visit		
<5 minutes	77%	80%
5–15 minutes	8%	16%
16–30 minutes	8%	4%
31–45 minutes	8%	0%
>45 minutes	0%	0%
Extra time required for clinic assessments		
<5 minutes	100%	94%
5–15 minutes	0%	6%
16–30 minutes	0%	0%
31–45 minutes	0%	0%
>45 minutes	0%	0%
Issues with a particular clinic assessment		
No problems	92%	94%
One or more assessments	8%	4%
Entire visit	0%	2%
Specific clinical assessments with issues		
Vitals	0%	0%
Medications	0%	0%
N-FOG-Q	0%	0%
UPDRS	8%	4%
TUG	8%	0%
Audio-video quality clinical assessment		
Great	69%	60%
Video a little slow	0%^#^	30%
Video quality mixed	15%	6%
Video details barely visible	0%	4%
Video dropping connection	0%	0%
No audio	0%	0%
Audio-video mismatch	0%	4%
Audio only, no video	0%	0%
Audio by telephone	0%	0%
Barely audible	0%	0%
Extra time required to setup research visit		
<5 minutes	100%	86%
5–15 minutes	0%	14%
16–30 minutes	0%	0%
31–45 minutes	0%	0%
>45 minutes	0%	0%
Extra time required to for research assessments		
<5 minutes	77%	58%
5–15 minutes	8%	18%
16–30 minutes	8%	10%
31–45 minutes	0%	2%
>45 minutes	8%	12%
Issues with a particular research assessment		
No problems	67%	78%
One or more assessments	33%	20%
Entire visit	0%	2%
Specific research assessments with issues		
MoCA-any component	15%	14%
MoCA-visuospatial	15%	10%
MoCA-other	15%	8%
Handwriting	8%	0%
Speech	8%	2%
PDQ-39	15%^#^	0%
RBD-ESS	15%^#^	2%
Post-visit survey	15%^#^	0%
Audio-video quality research assessments		
Great	77%	62%
Video a little slow	8%	20%
Video quality mixed	8%	8%
Video details barely visible	0%	4%
Video dropping connection	0%	2%
No audio	0%	0%
Audio-video mismatch	0%	6%
Audio only, no video	0%	0%
Audio by telephone	0%	6%
Barely audible	0%	2%

*p* < 0.05 by *Mann-Whitney *U*-Test or ^#^Chi-square test. MoCA = Montreal Cognitive Assessment; N-FOG-Q = New Freezing of Gait Questionnaire; PDQ = Parkinson’s disease quality of life scale; RBD = REM sleep behavior disorder; TUG = Timed-up-and-go test; UPDRS = Unified Parkinson’s disease Rating Scale.

## Discussion

In this pilot study, we enrolled PwPD residing in a predominantly rural state in a telehealth-based study utilizing clinic-to-clinic video visits at regional centers located close to the participant’s residence. We compared these results to Phase 1 of the same study, which included participants who performed telehealth visits at home only due to the COVID-19 pandemic. Despite the small number of participants at the regional centers, there are still several important findings from this pilot study. Our data suggests that lower socioeconomic status participants were as willing to participate in future telehealth research studies as people with higher income distributions who had the technological capabilities to participate in an at-home telehealth visit. The participants who performed visits at regional centers had greater disease burden, worse quality of life, and were more reliant on their children for transportation to clinic visits compared to those who were able to participate from home. Lastly, we found that satisfaction with the telehealth visits was high despite the provider interaction being over a computer screen, and less of the participants at the regional centers reported a preference for in-person visits. It is possible however that some of these findings are due to small participant numbers and selection bias, with more people enrolling in the study during the peak of the COVID pandemic for at-home visits who had higher incomes and better control of their PD symptoms.

Our cohort of PwPD who agreed to participate in the regional center telehealth visits had several important characteristics. Firstly, their lower socioeconomic status, based on their ADI scores, both within the state of Arkansas and nationally, disagrees with the notion that people from lower socioeconomic backgrounds don’t participate in research. As these participants enrolled after the peak of the pandemic and were seen in an in-person setting at a regional clinic (albeit by the MD via telemedicine), it is less likely that they enrolled in a research visit to be able to access care. It also suggests that they are not opposed to using technology for clinical care if they have a means to access such technology. Despite having a more socioeconomically diverse population, most of our participants were Caucasian, and better strategies to increase participation from ethnically diverse populations are still needed. However, employing methods to make research participation easier for PwPD, such as research visits at local regional centers utilizing a research network, could increase participation from a wider socioeconomic group. The only non-Caucasian participant (African American) was enrolled through the regional center arm of the study.

The regional center participant group had a higher disease burden including greater UPDRS scores, lower MoCA scores, and slower walking speeds, and this was subjectively reflected in worse quality of life scores. As the participants in both groups had similar disease duration, this difference could be related to decreased access to care, leading to undertreated disease. We cannot exclude the possibility that this decreased access to care was related to the COVID pandemic or a sampling bias due to the small number of participants. However, irrespective of the cause of the decreased access to care, in support of the idea that we enrolled a population with decreased access, there was a trend towards lower daily levodopa treatment doses in the regional center participants than the at-home participants (596 vs 662 mg daily levodopa, respectively). Overall socioeconomic status of participants only showed a weak association with MoCA scores and quality of life scores, but a stronger association with walking speed (Fig. 3). The regional center cohort that enrolled in our study was a population of PwPD who would benefit from greater access to clinical care. Future studies monitoring PwPD using longitudinal telehealth follow-up visits at regional centers are needed to determine if the disease metrics in this population could be improved with such a care model.

Access to care in a rural area, such as is the case for the majority of Arkansas, is difficult. Participants from home lived on average 60 miles from UAMS, while regional center participants lived on average 115 miles from UAMS. The travel time saved was almost 3 hours to perform visits at the local regional center compared to driving to UAMS. This difference could be related to greater recruitment of participants living closer to UAMS for at-home visits during the pandemic, or other temporal factors and selection bias as noted above. However, both groups of participants were still equally reliant on their children for transportation to visits. Providing easier access to care could make it easier for PwPD to obtain support for their clinical visits, thereby increasing the potential for more frequent visits if needed.

This study also provides another validation of remote administration of the modified UPDRS and MoCA in a small cohort [30-34]. Future incorporation of properly validated inexpensive and reliable sensors for remote objective evaluation of limb bradykinesia and gait [35-37] in rural and underserved areas could further extend our results.

While we did not target recruitment efforts towards enrollment of MUA participants in this study, 77% of the regional center participants resided in MUAs compared to 40% of the at-home participants, although again sampling bias due to the small cohort could account for any group differences. The quality of videoconferencing was subjectively a little slower in the at-home group which is one advantage of utilizing regional centers with higher bandwidth internet connectivity. However, surprisingly, there was still some variability in quality even at the regional centers. One important point to note was that the visits took over half an hour longer to complete at the regional centers than in the participants at home, despite the assessments being the same. One possibility for this time difference could be that participants at the regional centers were less familiar with technology, requiring more assistance to complete the questionnaire which were REDCap survey-based instruments requiring selection of the responses by the participants. This will be important to delve into in more detail and determine which components of the visit took longer as it may impact the costs of clinical care delivery using this modality.

Only 1 participant from the regional centers reported a preference for in-person visits, despite being asked to fill out the questionnaire after a 2-hour visit, instead of a typically 30-minute in-person visit. We also previously reported that approximately 30% of at-home participants who preferred in-person visits had a higher income distribution compared to those who did not report a preference for in-person visits [5]. Taken together, these findings suggest that lower socioeconomic status did not imply a hesitancy to telehealth-based visits and that providing a means to access the technology closer to home could overcome any potential digital divide. A hub-and-spoke network-based model utilizing local visits for routine care and access to advanced services present only at the hub center could be envisioned [6].

There are some limitations to the current pilot study. Sampling bias due to the small number of participants in the regional centers group could account for some of the differences we saw between the in-home and regional center cohorts. The two cohorts were also enrolled at different periods of time and were impacted by the COVID pandemic, with in-home participants recruited during the peak of the pandemic and regional center participants recruited when clinics were starting to open again. This could also impact group comparisons. We were not able to perform a cost comparison of the different visit types either in relation to direct costs to patients, insurance providers, or hospital and clinic networks to determine whether costs related to decreased travel would lead to overall reduction in out-of-pocket costs for patients. For greater adaptation of such a model, this would be important. Additionally, due to the number of disease features that we measured in our participants and the potential false discovery rate of 5%, caution should be taken to not overinterpret any statistical group differences.

In summary, we show that clinic-to-clinic telemedicine visits can be conducted in PwPD and can be incorporated into research studies in a population residing in medically underserved areas, with low socioeconomic status and possibly greater disease severity. These results provide preliminary support for a hub-in-spoke model to improve access to care for PwPD who otherwise would not have had access to the technology needed to perform home-based visits. Longitudinal studies to evaluate the ability to improve quality of life for such people in the future would be beneficial.

## Supporting information

Virmani et al. supplementary material Virmani et al. supplementary material

## Supplementary material

The supplementary material for this article can be found at https://doi.org/10.1017/cts.2024.498.
