# Supplementary material for: Feasibility of regional center telehealth visits utilizing a rural research network in people with Parkinson’s disease
Source: J Clin Transl Sci. 2024 Mar 25;8(1):e63. doi: 10.1017/cts.2024.498 (PMC11036429; doi:10.1017/cts.2024.498)
Supplement: Virmani et al. supplementary material [file S2059866124004989sup001.docx]

| Supplementary Table 1: Analysis on trimmed voice samples | | |
| --- | --- | --- |
|  | Regional center participants (n=9) | At-home participants (n=40) |
| *Ahh* sound duration (s) | 3.2 ± 1.3 | 3.6 ± 1.5 |
| *Ahh* sound f0 mean (Hz) | 180.3 ± 45.7 | 183.3 ± 44.9 |
| *Ahh* sound f0 standard deviation (Hz) | 8.8 ± 10.4 | 8.9 ± 14.3 |
| *Ahh* sound local Jitter (%) | 0.7 ± 0.4 | 0.6 ± 0.3 |
| *Ahh* sound local Shimmer (%) | 6.4 ± 3.7 | 6.7 ± 2.5 |
| *Ahh* sound HNR (dB) | 17.8 ± 4.2 | 18.1 ± 3.4 |
